# Merkel Cell Carcinoma in an Elderly Male With Extensive Local Disease

**DOI:** 10.7759/cureus.48001

**Published:** 2023-10-30

**Authors:** Robert Colef, Nfn Kiran, Leslie Mescallado, Fanyi Kong, Shahbaz Khan

**Affiliations:** 1 Pathology and Laboratory Medicine, Staten Island University Hospital, Northwell Health, New York, USA; 2 Gastrointestinal, Hepatobiliary and Transplant Pathology, Indiana University School of Medicine, Indianapolis, USA; 3 Hematopathology, Northwell Health, New York, USA; 4 Pathology, University of Oklahoma Health Sciences Center, Oklahoma City, USA

**Keywords:** elderly male, metastasis, excisional debridement, sun-exposed skin, neuroendocrine carcinoma, skin malignancy, merkel cell carcinoma of unknown primary

## Abstract

Merkel cell carcinoma (MCC) is a rare, highly aggressive neuroendocrine carcinoma of the skin. It is often found in the sun-exposed skin areas of elderly individuals of Caucasian descent. MCC has a tendency for local recurrence and the potential to invade nearby lymph nodes and spread to distant sites in the body. Here, we present the case of an 83-year-old male with a history of multiple comorbidities, including congestive heart failure, obesity, hypertension, benign prostatic hyperplasia, and sarcoidosis, who presented with a slow-growing, fungating lesion on his left lower leg. Histopathological examination revealed MCC with extensive necrosis and involved resection margins. Additional skin lesions on the left knee were confirmed to be MCC. Follow-up CT scans showed lymphadenopathy and a femoral lesion. The patient was deemed a poor candidate for resection and placed on immunotherapy treatment. The low incidence rate and indistinct clinical manifestations of MCC make a conclusive diagnosis dependent on examining histological features and immunohistochemical markers through a lesioned biopsy or resection. Due to the aggressive nature of MCC and the tendency for asymptomatic and painless lesions to escape notice, it is important to raise awareness about this condition. This will lead to earlier detection and intervention, potentially enhancing patient survival rates.

## Introduction

Merkel cell carcinoma (MCC) is a rare, aggressive skin cancer with a generally unfavorable prognosis. Initially described in 1972 as a relatively slow-growing disease, current research indicates a more aggressive course, marked by a rising incidence, and a significantly lower survival rate compared to melanoma, with a relative cumulative survival rate of 60.6% for MCC versus 94.7% for melanoma after one year and 40.2% for MCC versus 83.7% for melanoma after 10 years [1-4]. Recent findings indicate that these tumors stem from multipotent stem cells that demonstrate both epithelial and neuroendocrine characteristics during malignant transformation [5-7]. The annual worldwide incidence of MCC varies, ranging from 0.13 to 1.6 cases per 100,000, with more than nine out of 10 people diagnosed with MCC being Caucasian and older than 50 years old, with males having a higher incidence rate and a lower 10-year relative survival rate than women [8,9].

While the precise pathogenesis of MCC remains incompletely understood, several factors contribute to its development, including advanced age, cumulative ultraviolet exposure, immunosuppression, and infection with the Merkel cell polyomavirus (MCPyV) [10]. Clinically, MCC often presents as a fast-growing, painless, firm, raised, dome-shaped nodule with a varied appearance that is often red or violet on sun-exposed skin. Early-stage tumors are frequently misdiagnosed as benign skin conditions, such as cysts, lipomas, or pyogenic granulomas. Recognizing MCC at an early stage is crucial, as the five-year survival rate is 79% for Stage IA but drops significantly to only 18% once metastasis (Stage IV) occurs [11]. Maintaining a high level of clinical suspicion and achieving an early diagnosis can potentially improve survival rates.

## Case presentation

An 83-year-old male presented to the clinic with a wound-like lesion on his left lower leg near the ankle, which had slowly been growing over the course of a year, eventually reaching a size of 5 x 5 cm. The lesion developed hypertrophic granulation tissue and assumed a fungating appearance. His medical history included congestive heart failure, obesity, hypertension, benign prostatic hyperplasia, and sarcoidosis, diagnosed in his 30s. He had a history of smoking but had stopped in the distant past.

**Figure 1:**
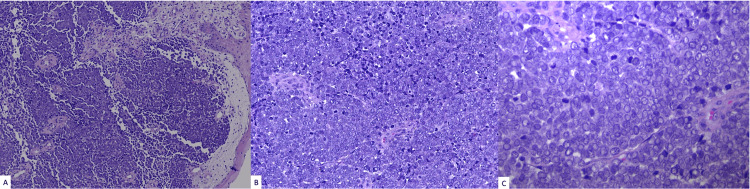
The tumor appears as a diffuse proliferation (a. hematoxylin and eosin (H&E) stain; 100×) of tightly packed small round blue cells (b. H&E; 200×) that exhibit scant pale cytoplasm, enlarged irregular nuclei with coarse granular chromatin, and numerous mitotic figures (c. H&E; 400×).

Excisional debridement was performed. Histologic examination of the skin resection revealed small, round, basophilic cells, uniform in size with scant cytoplasm and with vesicular nuclei, stippled chromatin, and indistinct nucleoli (Figure 1).

**Figure 2:**
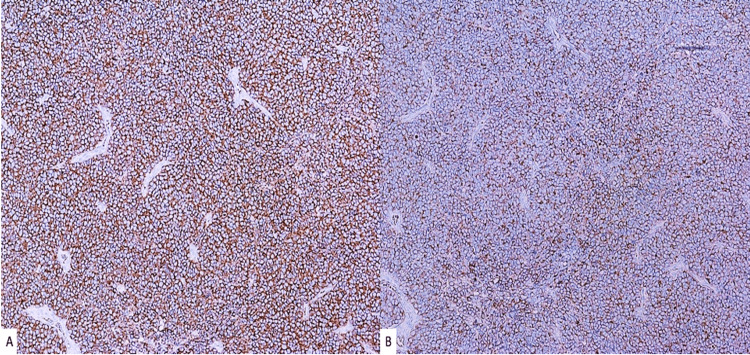
Immunohistochemical profile of Merkel cell carcinoma lesion showing positivity for (a) synaptophysin (100×) and (b) chromogranin A (100×).

**Figure 3:**
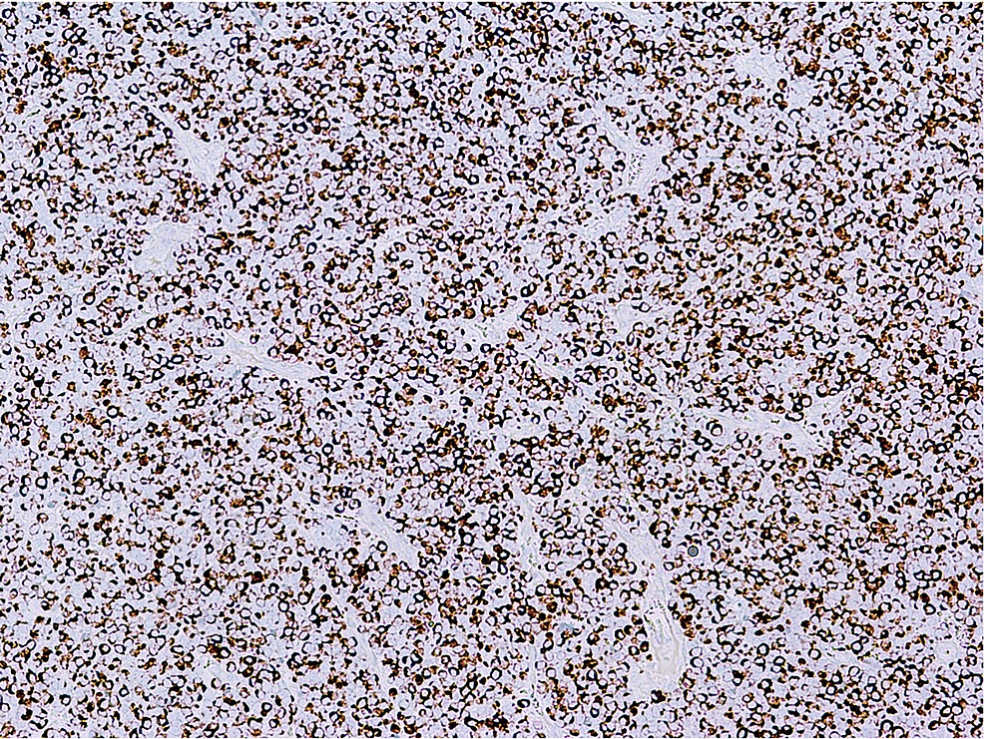
CK20, perinuclear dot pattern, (100×).

**Figure 4:**
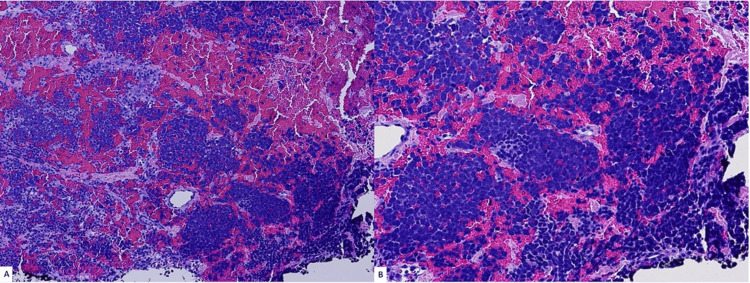
Left knee skin lesions punch biopsy showing extensive Merkel cell carcinoma with a similar diffuse pattern of tightly packed small round blue cells as in the wound lesion (a. hematoxylin and eosin (H&E); 100×). The tumor cells appear to involve the black-inked deep resection margin (b. H&E; 200×).

Extensive necrosis and numerous mitotic figures were also observed. Immunohistochemical (IHC) staining was performed and demonstrated that the tumor cells were positive for synaptophysin, chromogranin A (Figure 2), and CK20 (Figure 3), and negative for SOX-10, CK7, TTF-1, LCA (CD45), CD3, CD20. The Ki-67 proliferation index was greater than 95%. The features identified on histology and IHC were consistent with an MCC diagnosis, which measured 4.5 cm (pT2) in the greatest dimension. Both deep and peripheral margins were positive for carcinoma and showed necrosis. Lymphovascular invasion was also identified. There were an additional three skin lesions on the patient’s left knee, which had been present for six months. They were biopsied and confirmed to be positive for MCC (Figure 4).

**Figure 5:**
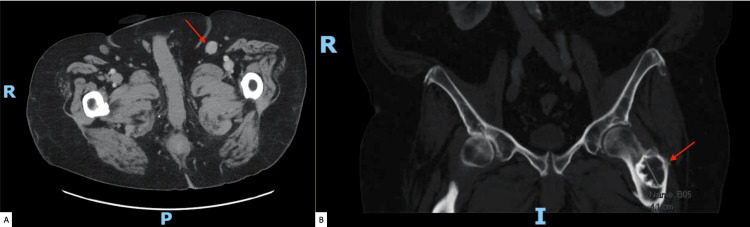
CT scan of the abdomen and pelvis reveals a prominent left groin lymph node measuring 1.8 cm (a) and a 4.1 cm lesion in the proximal left femur (b), both highlighted by a red arrow.

A subsequent abdomen and pelvis CT demonstrated a prominent left groin lymph node measuring up to 1.8 cm and a 4.1 x 3.6 x 2.4 cm lesion in the proximal left femur (Figure 5), raising suspicion of metastasis.

The patient was deemed a poor surgical candidate, making it challenging to pursue aggressive diagnostic procedures. Although programmed cell death protein 1 and programmed death-ligand 1 were not performed, given the patient’s medical issues, immune checkpoint inhibitor monotherapy was initiated with nivolumab administered intravenously at a dose of 480 mg every 28 days, with possible lesion resections in the future. At interval follow-up, the patient showed possible lesion sites on the left knee and shin decreasing in size and no new or developing lesions.

## Discussion

Originally documented in 1972, MCC is a rare neuroendocrine skin carcinoma [4,12]. It exhibits a significant predilection for fair-skinned individuals, comprising approximately 95% of cases, while its occurrence is less common among individuals of Asian, Native American, or African descent [13]. Data from the Surveillance, Epidemiology, and End Results program spanning from 1973 to 2006 revealed that Caucasians constituted the majority of MCC cases, accounting for 94.9%, whereas African Americans represented only 1% of the patient population [14]. The precise origin of MCC remains a subject of debate, with recent research suggesting that MCC may originate from pluripotent cutaneous stem cells [7].

MCC poses a formidable challenge due to its aggressive nature, substantial mortality rates, and MCPyV infection and immunosuppression associations. Even after radical surgical procedures, MCC frequently exhibits in situ relapse and metastasis to regional lymph nodes and distant sites such as skin, bone, hepatic, and lung metastasis [15].

Given the indistinct clinical characteristics of MCC, its diagnosis often hinges on pathological examination. Histologically, these tumors are characterized by small, round basophilic cells of uniform size, featuring scant cytoplasm, a high N/C ratio, vesicular nuclei with stippled chromatin, indistinct nucleoli, abundant conspicuous mitotic figures, and apoptotic bodies. MCC cases associated with a poorer prognosis include those with features of increased vascular proliferation, lymphovascular invasion, increased mitotic figures, smaller cell size, tumor size greater than 2 cm, male sex, age greater than 60 years, immunosuppression, and location on the lower extremities [16-19]. Involvement of the regional lymph nodes dramatically decreases the survival rates from 88% to 50%, and lymph node involvement occurs in 50% to 70% of all patients within two years of the lesion initially appearing [20].

The diagnostic process for MCC can be particularly difficult due to the atypical histomorphological characteristics of MCC cells and the fact that MCC may be confused with other skin neoplasms, including basal cell carcinoma, cutaneous lymphoma, melanoma, small cell lung cancer, or Ewing’s sarcoma [15]. IHC staining functions as a pivotal tool in distinguishing MCC from other tumors. Notably, MCC tumor cells stain positively for neuroendocrine markers such as synaptophysin and chromogranin A and epithelial markers such as CK20, often demonstrating a characteristic perinuclear dot pattern. In the context of our case, these tests were complemented by staining for LCA (CD45), TTF-1, SOX-10, CD3, and CD20, which are typically negative in most MCC cases and aid in establishing a comprehensive differential diagnosis.

## Conclusions

This case underscores the complex clinical presentation of MCC in an 83-year-old patient, characterized by multiple adverse prognostic factors such as lymphovascular invasion and the presence of multiple discontinuous lesions in the lower extremity. Potential metastasis to a groin lymph node and the femur further complicates this case and emphasizes vigilant monitoring and ongoing evaluation. The patient’s advanced age and preexisting comorbidities, including congestive heart failure, obesity, hypertension, benign prostatic hyperplasia, and sarcoidosis, significantly challenged the feasibility of surgical intervention. This case demonstrates the importance of enhancing awareness surrounding MCC, particularly within susceptible populations. This tumor’s aggressive, fast-spreading nature is highlighted in this case with an initial lesion presenting one year before diagnosis with additional, separate lesions seen six months after the initial lesion presented. The case is complicated by the possibility of lymph node and bone metastasis in a patient who was evaluated not to be a surgical candidate. Hence, early detection remains pivotal in augmenting patient outcomes. Disseminating this case promotes a deeper comprehension of the multifaceted nature of MCC and urges healthcare professionals to maintain heightened vigilance when evaluating individuals, especially those harboring risk factors. Timely diagnosis and intervention remain the key to improving clinical outcomes in the management of this unusual malignant neoplasm.
